# Markers for the identification of late breast cancer recurrence

**DOI:** 10.1186/s13058-015-0516-0

**Published:** 2015-01-27

**Authors:** Ivana Sestak, Jack Cuzick

**Affiliations:** Center for Cancer Prevention, Wolfson Institute of Preventive Medicine, Queen Mary University of London, Charterhouse Square, London, EC1M 6BQ UK

## Abstract

Postmenopausal women with early breast cancer are at an ongoing risk of relapse, even after successful surgery and treatment of the primary tumor. The treatment of breast cancer has changed in the past few years because of the discovery of prognostic and predictive biomarkers that allow individualized breast cancer treatment. However, it is still not clear how to identify women that are at high risk of a late recurrence. Clinical parameters are good prognostic markers for early recurrence, but only nodal status and, to a lesser extent, tumor size have proven to be strong prognostic markers for late recurrence. Multi-gene signatures have become widely used for the prediction of overall recurrence risk and tailoring administration of adjuvant chemotherapy, but only a few have been shown to be prognostic for late (distant) relapse. There is a need to accurately identify women who may benefit from extended endocrine therapy but also those who may be spared any additional treatment. Recent results from large clinical trials have shown that the research is going in the right direction, and these results might help to optimize extended endocrine therapy for patients with early breast cancer. However, further research is needed to select individual biomarkers or multi-gene signatures that offer identification of late recurrence specifically and thus justify routine use of these tests in the clinical setting.

**Electronic supplementary material:**

The online version of this article (doi:10.1186/s13058-015-0516-0) contains supplementary material, which is available to authorized users.

## Introduction

Breast cancer is the most common cancer in women, and over 1.6 million cases are diagnosed annually [1]. It is a highly heterogeneous disease with respect to clinical and molecular characteristics. Both adjuvant endocrine therapy and chemotherapy, after initial surgery, have proven to be highly effective methods to reduce the risk of disease recurrence, preventing both local and distant metastasis [2] and reducing mortality. Despite the proven benefits of adjuvant endocrine therapy in women with hormone receptor positive breast cancer, relapses still occur even after initial treatment with endocrine therapy for 5 years. Adjuvant chemotherapy has also proven to be effective in reducing the risk of recurrence within the first 5 years after diagnosis [2], but little is known about its effect on long-term outcome.

For women with HR-negative disease, the risk of recurrence is confined mostly to the first 5 years after diagnosis and relapse rates fall rapidly thereafter [3,4]. However, women with HR-positive tumors remain at risk for late recurrences, and the annual rate is in excess of 2% for at least 15 years, even after 5 years of tamoxifen therapy [5]. The ATLAS (Adjuvant Tamoxifen: Longer Against Shorter) and aTTom (Adjuvant Tamoxifen: To Offer More?) trials have reported their findings, and results show that extended tamoxifen therapy (5 additional years) in women with early-stage breast cancer reduces the risk of late recurrences [6]. Similar findings were observed for aromatase inhibitors, which have been shown to reduce the risk of late relapse if treatment was extended beyond the initial 5 years [7,8]. However, there is a need to identify those women at high risk of recurrence and who will benefit most from these extended therapies. Thus, the benefits of extended treatment must be weighed against potential side effects of prolonged therapy for each individual patient. In this article, we will review the evidence for the use of clinicopathological factors, individual biomarkers, and multi-gene/molecular scores for the prediction of late recurrence, especially distant recurrence.

## Clinicopathological parameters

Tumor size, stage, and nodal involvement are routinely used to estimate the likelihood of breast cancer recurrence and are relevant for both estrogen receptor (ER)-positive and -negative cancer. These clinical parameters are useful for predicting recurrence in the first 5 years after diagnosis [9]. Distant recurrence has been associated with large tumor size, poorly differentiated disease, and nodal involvement [10], and these factors are believed to be correlated also with late metastasis. However, only a few studies have investigated the utility of these clinical factors in this setting. The Netherlands Cancer Institute has studied 252 breast cancer patients with clinical data, long-term follow-up, and treatment details [11] and found that, of the clinical parameters, only nodal involvement was a significant prognostic factor for late metastasis. In the large translational Arimidex, Tamioxiden Alone or in Combination (transATAC) trial, we confirmed the role of nodal status but also found tumor size to be an independent prognostic factor for early and late distant recurrence, whereas grade was predictive only in the first 5 years after diagnosis (Table 1) [9]. These two findings are in agreement with previously reported results that nodal involvement is the dominant feature predicting disease-specific outcome [4]. Clinicopathological factors still have different values for the identification of women at high risk of late recurrence, and better discrimination is desirable. Many women with node-positive disease receive chemotherapy, but some may be over-treated and may be at a sufficiently low risk of recurrence 5 years after diagnosis. This raises the question of the duration of endocrine treatment, and it is not yet clear which women will benefit most from extended therapy.

**Table 1:** **Individual clinical and immunohistochemical markers for the prediction of early versus late recurrence in transATAC**

	**0-5 years**		**5-10 years**	
	**(number of all recurrences = 83)**		**(number of all recurrences = 107)**	
	**Univariate**	**Multivariate**	**Univariate**	**Multivariate**
	LR-χ^2^	LR-χ^2a^	LR-χ^2^	LR-χ^2a^
	(*P* value)	(*P* value)	(*P* value)	(*P* value)
Nodal status (negative versus positive)	17.24	7.60	30.80	18.99
	(<0.0001)	(0.006)	(<0.0001)	(<0.0001)
Tumor size (≤2 versus >2 cm)	28.95	10.96	25.40	13.03
	(<0.0001)	(0.0009)	(<0.0001)	(0.0003)
Grade (well/moderate versus poor)	21.88	5.22	1.74	-
	(<0.0001)	(0.02)	(0.2)	
ER_10_	12.17	5.51	1.65	-
	(0.004)	(0.02)	(0.2)	
PgR_10_	18.81	6.46	1.45	-
	(<0.0001)	(0.01)	(0.2)	
Ki67	17.90	6.52	9.04	2.14
	(<0.0001)	(0.01)	(0.003)	(0.1)
HER2 (negative versus positive)	15.45	3.18	0.04	-
	(<0.0001)	(0.07)	(0.8)	

^a^For addition to model containing all other factors that were significant in the univariate model. ATAC, Arimidex, Tamoxifen, Alone or in Combination; ER, estrogen receptor; HER2, human epidermal growth factor; LR, likelihood ratio; PgR, progesterone receptor. Table was adapted from [9].

## Cancer-related genes

The identification of markers for the prediction of breast cancer has been widely investigated, specifically for the identification of biomarkers for early breast cancer diagnosis. The evaluation of ER, progesterone receptor (PgR), Ki67, and human epidermal growth factor (HER2) is common in clinical practice for prognostic purposes and treatment decisions. However, laboratory variability in Ki67 scoring is well known and therefore this biomarker is not ideal for clinical decision making [12]. Other markers, such as B-cell lymphoma 2, androgen receptor, epidermal growth factor, phosphatase and tensin homolog, and *PIK3CA*, have been investigated for their prognostic value in breast cancer. Tumors with mutations in *PIK3CA* have been shown to be associated with lower recurrence and mortality rates in the late time period [13,14]. Liu and colleagues [15] analyzed predictors of late relapse in early-stage ER-positive breast cancer, in which differences in the primary tumor tissue in patients with distant relapses occurring early (less than 3 years) versus late (after 7 years) have been compared. A set of genes was identified that were prognostic specifically for early relapse (*CALM1*, *CALM2*, *CALM3*, *SRC*, *CDK1*, and *MAPK1*), but they also identified genes that appear to predict late relapse (*ESR1*, *ESR2*, *EGFR*, *BCL2*, and *AR*). High expression levels of *BCL2* have also been shown to be a good predictor of late breast cancer recurrence in a subset of 73 patients with primary breast cancer [16]. *TP53* gene mutation is very frequently found in breast cancers [17]. High expression of p53 in HR-positive tumors has been associated with risk of both early and late recurrence [18]. Furthermore, Bianchini and colleagues [19] investigated a mitotic kinase score (average expression of 12 kinases) and an estrogen-related score (four genes) and observed that women who recurred late (after 5 years) had highly proliferative and high estrogen-sensitive tumors. In contrast, in the transATAC trial, in which ER, PgR, Ki67, and HER2 as measured by immunohistochemistry were analyzed for their prognostic value for late distant recurrence, no prognostic information for late recurrence was observed for any of these four markers (Table 1) [9]. It remains to be seen whether these various genes can prospectively predict late (distant) recurrence and be implemented in the clinical setting.

## Molecular scores

Over the past decade, many molecular markers have been developed for the prediction of recurrence risk. Most of them are good prognostic markers during the first 5 years after diagnosis (Table 2). All of these markers have in common that they evaluate the patient’s individual risk of recurrence (ROR) by combining expression profiles of a panel of cancer-related genes. However, they need to be combined with traditional clinicopathological factors to make use of all available information on prognosis.

**Table 2:** **Summary of multi-gene/molecular scores for the prediction of recurrence**

**Score**	**Abbreviation**	**Details**	**Reference**
MammaPrint	MammaPrint	70 gene-based expression profile using DNA microarray. Fresh frozen material is used to perform analysis.	[20,21]
Genomic Grade Index	GGI	97 gene-based assay using DNA micro array. Fresh frozen material is used to perform the analysis.	[23,24]
Oncotype Dx Recurrence Score	RS	21 gene-based expression profile score using qRT-PCR (16 cancer genes, 5 housekeeping genes). FFPE blocks used to extract RNA.	[25]
Immunohistochemical Score 4	IHC4	Includes information on estrogen receptor (ER), progesterone receptor (PgR), Ki67, and HER2. Score developed on transATAC data. FFPE blocks used to extract RNA to perform IHC for ER, PgR, Ki67, and HER2.	[31]
Prosina Risk of Recurrence Score	ROR	50 gene-based expression profile score using qRT-PCR. FFPE blocks used to extract RNA to perform analysis on nCounter system.	[34]
Breast Cancer Index	BCI	Multi-gene assay using qRT-PCR. Combination of two biomarkers HOXB13/IL17BR (H/I) and molecular grade index (MGI). FFPE blocks used to extract RNA to perform analysis.	[38,53]
EndoPredict	EPclin	12 gene-based expression profile score using qRT-PCR (8 cancer genes, 4 housekeeping genes). FFPE blocks used to extract RNA to perform analysis.	[41]

ATAC, Arimidex, Tamoxifen, Alone or in Combination; FFPE, formalin-fixed paraffin-embedded; HER2, human epidermal growth factor; qRT-PCR, quantitative real-time polymerase chain reaction.

Currently, seven genomic assays are available for use in early-stage breast cancer (Table 2). All of these give an overall risk assessment of breast cancer recurrence and provide prognostic information not contained in clinicopathological factors. None of these signatures was specifically developed for predicting late (distant) recurrence; nevertheless, some of them have been investigated in this context, as summarized below.

## Current genetic tests for the prediction of recurrence

### MammaPrint

The MammaPrint (Agendia, Irvine, CA, USA) is a 70 gene-based molecular score, which was developed in a cohort of women who did not receive systemic therapy and with no long-term clinical outcome data [20,21]. The genes included in the MammaPrint are all related to the regulation of cell cycle, invasion, metastasis, proliferation, and angiogenesis and were retrospectively selected. This multi-gene score classifies women into low-risk (good prognosis) or high-risk (bad prognosis) groups according to their 10-year distant recurrence risk of less than 10% or more than 10%, respectively, but no continuous score is available for MammaPrint. The signature was validated in two studies in women with node-negative or node-positive disease and showed that the test predicted breast cancer recurrence accurately [20,21]. The predictive value of this test is being evaluated in the MINDACT (Microarray In Node-negative and 1-3 node-positive Disease may Avoid Chemo Therapy) trial where women are randomly assigned to chemotherapy plus endocrine therapy or endocrine therapy alone if the results between MammaPrint and Adjuvant Online! were discordant [22]. The MammaPrint has been evaluated in many patient subgroups, but the prognostic information added by this assay is confined to the first 5 years after diagnosis.

### Genomic grade index

The Genomic Grade Index (Ipsogen, now known as Qiagen Marseille, Marseille, France) is a 97 gene-based assay including mostly proliferation genes and was developed to refine the commonly used histological grade assessment (low, intermediate, and high). The assay classifies women into low- or high-grade groups and has been shown to better define tumor grade, patient prognosis, and breast cancer subtypes [23,24]. Again, this assay has been validated in many different patient subgroups, and the prediction of (distant) recurrence was limited to 5 years after diagnosis.

### Oncotype DX recurrence score

The 21 gene-based Oncotype Recurrence Score (Genomic Health, Redwood City, CA, USA) is a well-established multi-gene assay, which was developed to assess the risk of recurrence in women with HR-positive, node-negative breast cancer treated with tamoxifen [25]. The signature is based on 16 breast cancer-specific genes and five reference genes, including information on proliferation, estrogen, invasion, HER2, and other factors [25]. A mathematical algorithm was used to generate a continuous recurrence score (RS), with a higher RS corresponding to an increased risk of recurrence. The RS furthermore classifies women into low-risk (less than 18), intermediate-risk (18 to 30), and high-risk (more than 30) groups for recurrence. The RS has been validated and evaluated in a number of clinical trials and patient subgroups, and all results confirm the prognostic performance of the RS score for (distant) recurrence in the first 5 years after diagnosis [25-27].

The Oncotype RS has also been investigated in postmenopausal women receiving aromatase inhibitors as adjuvant treatment. In the transATAC trial [28], the RS has been evaluated in 1,125 postmenopausal women who were chemotherapy-free and who received either tamoxifen or anastrozole alone for 5 years. In this patient group, the RS was shown to add significant prognostic information for distant recurrence, independently of clinicopathological factors and Adjuvant Online!. The results furthermore confirmed that a combination of molecular predictors, as found in the RS, and clinical factors would provide a good prognostic tool for clinicians and oncologists [28].

The predictive value of the Oncotype RS has not been evaluated in the transATAC trial, but other studies have shown that women with a low RS will benefit little if at all from additional chemotherapy [26,27,29,30]. Nevertheless, the RS analysis in the transATAC trial confirmed indirectly that those with a very low RS, even with one to three positive nodes, will not benefit from additional chemotherapy.

The prognostic performance of the RS for late distant recurrence was furthermore evaluated in the transATAC trial [9] and compared with clinical factors and other molecular scores. The Oncotype RS added significant prognostic value in the first 5 years after breast cancer diagnosis but failed to be substantially predictive of late distant recurrence in the overall population in years 5 to 10 when adjusted for clinical parameters. The RS was more prognostic for late metastasis in women with HER2-negative disease and added little prognostic value for those with node-positive disease [9]. The RS was least prognostic for late distant recurrence when compared with other multi-gene scores, such as the PAM50 ROR score or the immunohistochemistry 4 (IHC4) score [31] (ER, PgR, Ki67, and HER2). The results of this analysis showed that the RS is not a strong candidate for the prediction of late distant recurrence.

## Prosigna PAM50 risk of recurrence

The PAM50 ROR (NanoString Technologies, Seattle, WA, USA) score is based on a 50-gene test, which was developed to identify intrinsic breast cancer subtypes [32,33]. The ROR is derived from an expression profile of the 50 genes and includes information on tumor size as well. The ROR score has been validated in women with node-negative or node-positive disease [32,33] and has been shown to classify women into low- or high-risk groups and added prognostic information beyond that of clinical or IHC4 factors.

In the transATAC trial, the PAM50 has been evaluated for the ability to add prognostic information for distant recurrence beyond that of clinical factors [34]. Furthermore, the prediction of distant recurrence in years 0 to 10 was compared with the Oncotype RS and IHC4 as well. The results showed that the ROR added significant prognostic information beyond that of clinical parameters in all patients and furthermore in all subgroups as well. In addition, it was shown that the ROR was more predictive of distant recurrence overall than the Oncotype RS and categorized more patients into the high-risk group and fewer into the intermediate-risk group than the RS, indicating better discrimination of risk groups.

The ROR score was also evaluated in the ABCSG-8 (Austrian Breast and Colorectal Cancer Study Group 8) trial, in which postmenopausal women with early breast cancer were randomly assigned to receive tamoxifen or anastrozole for 5 years [35]. In this large analysis, the ROR score added significant prognostic value beyond that of clinical parameters for distant recurrence in the overall population and all subgroups. It also confirmed the better discrimination between low- and high-risk groups in all subgroups.

The ROR score was further investigated for the prediction of late distant recurrence in the transATAC trial [9]. In this context, the ROR score was the most prognostic overall and in all patient subgroups, when compared with the Onctoype RS and IHC4, and reclassified more women into the low- or high-risk group than the other two tests. This was the first analysis to show that the ROR score, though developed to predict overall recurrence, added significant prognostic information for late distant recurrence. In a combined analysis of the transATAC and ABCSG-8 trials, the ROR score was investigated for the prediction of specifically late distant recurrence [36]. Two thousand one hundred and thirty-seven postmenopausal women who did not have a recurrence in the first 5 years after diagnosis were included in the analysis. In both the univariate and bivariate analyses (adjusted for clinical parameters), the ROR score added significant prognostic information for late distant recurrence in all patients and was more predictive in node-negative and node-negative/HER2-negative patients than clinical factors alone [36]. The results of this analysis indicated that the ROR score is able to identify women who are at sufficiently low risk of late distant recurrence, even if they have node-positive disease, and who might be spared additional endocrine therapy and therefore overtreatment.

### Breast cancer index

The Breast Cancer Index (BCI) (bioTheranostics, San Diego, CA, USA) is a gene expression module based on two components - the HOXB13/IL17BR (H/I) and the molecular grade index (MGI), which is a proliferation module. The continuous BCI score with a cubic component has been developed in tamoxifen-treated women with lymph node-negative disease and has been shown to be a good predictor for distant recurrence in this cohort [37]. A second risk model was developed by combining H/I and MGI into a linear, continuous risk score [38] and this model provided better significant prognostic information for distant recurrence in node-negative patients. Furthermore, the linear BCI stratified the majority of women as low risk for distant recurrence in years 0 to 5 and also in years 5 to 10 after diagnosis.

The BCI was further evaluated in the transATAC cohort in which both the cubic BCI and the linear BCI were investigated for the prediction of distant recurrence in the early and late follow-up period [39]. The results of this analysis showed that only the linear BCI as a continuous score was an independent strong predictor for distant recurrence in women with node-negative or HER2-negative/node-negative disease. As a categorical score, the linear BCI compared low-, intermediate-, and high-risk groups, and significant differences in distant recurrence rates at 5 years of follow-up were found for the high-risk group when compared with the low/intermediate-risk group (1.3% versus 5.6% versus 18.1%; *P* < 0.001). Conversely, in years 5 to 10 after diagnosis, the low-risk group had a recurrence rate of 3.5%, which was significantly lower when compared with the intermediate-risk (13.4%) and high-risk (13.3%) group (*P* = 0.001). To further evaluate whether both components of the BCI score predict for late distant recurrence, an exploratory analysis was performed to investigate the prognostic performance of each component separately. The results showed that, although both components added prognostic value in the first 5 years after diagnosis when adjusted for clinical parameters, only H/I also significantly predicted for late recurrence [39]. The finding that MGI is not prognostic for late distant recurrence is consistent with other results [9] in which proliferation-related factors, such as Ki67 or clinical tumor grade, are not prognostic for late distant recurrence. Overall, the BCI identifies women with node-negative disease at increased risk of late distant recurrence and might be used in the clinical setting to identify women who might benefit from further endocrine therapy. However, this signature has not been evaluated in women with node-positive disease.

### EndoPredict

The EndoPredict (EP) (Sividon, Cologne, Germany) has been developed for women with HR-positive/HER2-negative disease and includes information of eight cancer-related and three normalization genes [40]. The signature has been validated in a cohort of women from two large clinical trials (ABCSG-6/8), which were treated with adjuvant endocrine therapy only. Apart from the EP score, a second score, EPclin, has been developed, which combines the EP score with clinical information, such as nodal status and tumor size. Both signatures are available as continuous scores or as low-risk and high risk groups as defined by pre-specified cutoff points. In both validation cohorts, the EP was an independent predictor of distant recurrence beyond that of clinical variables. Furthermore, the EPclin provided additional prognostic information beyond that of clinicopathological parameters alone in women with HER2-negative breast cancer. In the same two cohorts, the EP and EPclin were specifically investigated for the prediction of late distant recurrence [41]. The results showed that both signatures are clearly associated with the prediction of late metastasis. The EPclin furthermore identified a subgroup of patients who have a very good prognosis after 5 years of endocrine therapy.

## Novel predictors

None of the multi-gene tests discussed above was specifically developed for the prediction of late recurrence, and there is great interest in new biomarkers for the prediction of these events. The ultimate goal of a new biomarker is that it specifically predicts for either early or late recurrence. Cheng and colleagues [42] identified a 51-gene signature from primary breast cancer tumors, which are particularly associated with late recurrence. Although this gene signature has not been validated yet, it suggests that novel biomarkers might help to identify women who might be at risk of late recurrence and therefore candidates for extended therapy.

A new area of great interest is the identification of circulating tumor cells (CTCs) in peripheral blood of patients with breast cancer (from either primary tumor or metastasis). CTCs have been linked to worse prognosis and early relapse in breast cancer and can be used for the identification of response to treatment [43]. Many studies have shown that CTCs in early breast cancer predict recurrence within the first 5 years after diagnosis [44-47]. An overview of all studies investigating CTCs for detection of metastatic breast cancer was recently published [48] (Table 3). CTCs thus far have been investigated mostly in the metastatic setting, and further research is needed for the early breast cancer setting.

**Table 3:** **Summary of ongoing clinical trials with circulating tumor cells in metastatic breast cancer**

**Trial**	**Study design**	**Reference**
STIC CTC METABREAST	1,000 hormone receptor-positive, metastatic breast cancer patients randomly assigned to either standard arm or CTC-driven arm, which dictates whether hormone therapy (5 CTCs <7.5 mL) or to chemotherapy (5 CTCs ≥7.5 mL) is administered. Trial aims to show non-inferiority of the CTC arm versus standard arm for progression-free survival.	[54]
SWOG 0500	Screening patients with metastatic disease and more than 5 CTCs (n = 610) and randomization between continuation of first-line therapy (CTC response, <5 CTC/7.5 mL) and switch to another chemotherapy regime (no CTC response, ≥5 CTCs/7.5 mL) (n = 120). Primary endpoint is improvement in overall survival in the CTC-driven arm.	[55]
CirCe01	304 women with metastatic disease starting with third-line chemotherapy will be randomly assigned between a CTC-driven arm and standard care arm. Patients in the CTC-driven arm change chemotherapy regimens according to their CTC counts during treatment. Primary endpoint is overall survival.	[56]
Treat CTC	Patients with HER2-negative breast cancer having completed chemotherapy and primary surgery and with at least 1 CTC/15 mL will be randomly assigned to an observation arm or to receive trastuzamab. Primary endpoint is detection rate of CTCs after 18 weeks between the two arms.	NCT01548677
DETECT III	Around 300 patients with metastatic and HER2-negative disease with detectable at least one HER2-positive CTC/7.5 mL will be randomly assigned to receive standard care (endocrine or chemotherapy or both) versus standard care plus lapatinib. Primary endpoint is progression-free survival.	NCT01619111

CTC, circulating tumor cell; HER2, human epidermal growth factor.

In addition, the detection of one or more CTCs in the first 5 years after diagnosis has been associated with late relapse in early breast cancer [49]. A potential advantage of CTCs is that their presence can be measured at several time points during disease from blood samples (‘liquid biopsies’) and thus give information on disease progression. However, it is still not clear what the best detection method for CTCs is, as these cells occur at a very low yield. Another research area that needs to be addressed is the correct time point of measuring CTCs for late relapse. Although it has been become clear that CTCs are a powerful marker for the prediction of early metastatic disease, not many studies have specifically addressed the use of CTCs for the identification of late relapse and this area of research needs further investigation.

## Conclusions

It is important to accurately identify women at high risk of late (distant) recurrence as some of them may be spared extended endocrine therapy whereas others may benefit from further treatment. Risk of distant recurrence and timing vary among patients with breast cancer. Clinicopathological parameters, specifically nodal status and tumor size, are well-established predictors for late recurrence in postmenopausal women with HR-positive breast cancer. However, it has become evident that molecular signatures improve the prediction of late relapse and can identify women who are at sufficiently low risk, even with node-positive disease, who do not warrant extended endocrine therapy. The ROR score, the BCI, and the EP score all have been shown to add significant prognostic information for late (distant) recurrence in postmenopausal women who are chemotherapy-free and these results indicate that these signatures may be helpful in the clinical setting.

However, the question of which molecular test is best for each individual patient needs further research. Although all of the signatures predict early and some of them late recurrence in a variety of patients, it has become apparent that tumor biology is not all that matters. Non-clinical baseline factors, such as age or body mass index, may influence the prognostication of these signatures [50] and furthermore may help to identify specific women who will benefit most from these tests.

The difficulty that all discussed molecular signatures have in common is that the information is derived from the primary tumor, assuming that driving forces for late recurrences are in these primary tumors. This might be true for early relapses but not necessarily for late recurrence. Dormant micrometastasis cells might survive for a long time in distant organs and undergo genetic changes, which might drive the recurrence but not be present in the primary tumor [51]. CTCs (micro-metastasis) may stay dormant in distant organs for many years, and genetic changes of these cells may arise and reflect differences in these cells that are distinctively diverse from those of the primary tumor [52]. Therefore, it remains a challenge to accurately classify primary tumors for the prediction of late recurrences.

Many molecular signatures have been evaluated and validated in large clinical trials, and most of them are officially approved for use in the clinic. None of the discussed signatures has been developed particularly for the prediction of late (distant) recurrence. Nevertheless, the results clearly show that some of them have great potential for the identification of women who are at high risk of developing a late recurrence, particularly those with node-negative disease. With the help of these signatures, tailored therapies and specific patient treatments can be optimized for late (distant) recurrence.

## Abbreviations

ABCSG: Austrian breast and colorectal cancer study group
ATAC: Arimidex, tamoxifen, alone or in combination
BCI: Breast cancer index
CTC: Circulating tumor cell
EP: EndoPredict
Epclin: EndoPredict plus clinical factors
ER: Estrogen receptor
H/I: HOXB13/IL17BR
HER2: Human epidermal growth factor
HR: Hormone receptor
IHC: Immunohistochemistry
MGI: Molecular growth index
PgR: Progesterone receptor
ROR: Risk of recurrence
RS: Recurrence score

## References

[1] Ferlay J, Soerjomataram I, Ervik M, Dikshit RP, Eser S, Mathers C, et al. GLOBOCAN 2012 v1.0, Cancer Incidence and Mortality Worldwide: IARC CancerBase No. 11 [Internet]. International Agency for Research on Cancer 2013. [http://globocan.iarc.fr/]

[2] Early Breast Cancer Trialists’ Collaborative Group (EBCTCG) (2005). Effects of chemotherapy and hormonal therapy for early breast cancer on recurrence and 15-year survival: an overview of the randomised trials. Lancet.

[3] Cheang MC, Voduc D, Bajdik C, Leung S, McKinney S, Chia SK, Perou CM, Nielsen TO (2008). Basal-like breast cancer defined by five biomarkers has superior prognostic value than triple-negative phenotype. Clin Cancer Res.

[4] Esserman LJ, Moore DH, Tsing PJ, Chu PW, Yau C, Ozanne E, Chung RE, Tandon VJ, Park JW, Baehner FL, Kreps S, Tutt AN, Gillett CE, Benz CC (2011). Biologic markers determine both the risk and the timing of recurrence in breast cancer. Breast Cancer Res Treat.

[5] Saphner T, Tormey DC, Gray R (1996). Annual hazard rates of recurrence for breast cancer after primary therapy. J Clin Oncol.

[6] Davies C, Pan H, Godwin J, Gray R, Arriagada R, Raina V, Abraham M, Medeiros Alencar VH, Badran A, Bonfill X, Bradbury J, Clarke M, Collins R, Davis SR, Delmestri A, Forbes JF, Haddad P, Hou MF, Inbar M, Khaled H, Kielanowska J, Kwan WH, Mathew BS, Mittra I, Müller B, Nicolucci A, Peralta O, Pernas F, Petruzelka L, Pienkowski T, Radhika R, Rajan B, Rubach MT, Tort S, Urrútia G, Valentini M, Wang Y, Peto R, Adjuvant Tamoxifen: Longer Against Shorter (ATLAS) Collaborative Group (2013). Long-term effects of continuing adjuvant tamoxifen to 10 years versus stopping at 5 years after diagnosis of oestrogen receptor-positive breast cancer: ATLAS, a randomised trial. Lancet.

[7] Jakesz R, Greil R, Gnant M, Schmid M, Kwasny W, Kubista E, Mlineritsch B, Tausch C, Stierer M, Hofbauer F, Renner K, Dadak C, Rücklinger E, Samonigg H, Austrian Breast and Colorectal Cancer Study Group (2007). Extended adjuvant therapy with anastrozole among postmenopausal breast cancer patients: results from the randomized Austrian Breast and Colorectal Cancer Study Group Trial 6a. J Natl Cancer Inst.

[8] Goss PE, Ingle JN, Martino S, Robert NJ, Muss HB, Piccart MJ, Castiglione M, Tu D, Shepherd LE, Pritchard KI, Livingston RB, Davidson NE, Norton L, Perez EA, Abrams JS, Cameron DA, Palmer MJ, Pater JL (2005). Randomized trial of letrozole following tamoxifen as extended adjuvant therapy in receptor-positive breast cancer: updated findings from NCIC CTG MA.17. J Natl Cancer Inst.

[9] Sestak I, Dowsett M, Zabaglo L, Lopez-Knowles E, Ferree S, Cowens JW, Cuzick J (2013). Factors predicting late recurrence for estrogen receptor-positive breast cancer. J Natl Cancer Inst.

[10] Castano Z, Tracy K, McAllister SS (2011). The tumor macroenvironment and systemic regulation of breast cancer progression. Int J Dev Biol.

[11] Mittempergher L, Saghatchian M, Wolf DM, Michiels S, Canisius S, Dessen P, Delaloge S, Lazar V, Benz SC, Tursz T, Bernards R, van’t Veer LJ (2013). A gene signature for late distant metastasis in breast cancer identifies a potential mechanism of late recurrences. Mol Oncol.

[12] Polley MY, Leung SC, McShane LM, Gao D, Hugh JC, Mastropasqua MG, Viale G, Zabaglo LA, Penault-Llorca F, Bartlett JM, Gown AM, Symmans WF, Piper T, Mehl E, Enos RA, Hayes DF, Dowsett M, Nielsen TO, International Ki67 in Breast Cancer Working Group of the Breast International Group and North American Breast Cancer Group (2013). An international Ki67 reproducibility study. J Natl Cancer Inst.

[13] Sanchez CG, Ma CX, Crowder RJ, Guintoli T, Phommaly C, Gao F, Lin L, Ellis MJ (2011). Preclinical modeling of combined phosphatidylinositol-3-kinase inhibition with endocrine therapy for estrogen receptor-positive breast cancer. Breast Cancer Res.

[14] Kalinsky K, Jacks LM, Heguy A, Patil S, Drobnjak M, Bhanot UK, Hedvat CV, Traina TA, Solit D, Gerald W, Moynahan ME (2009). PIK3CA mutation associates with improved outcome in breast cancer. Clin Cancer Res.

[15] Liu MC, Dixon JM, Xuan JJ, Riggins RB, Chen L, Wang C, Cho Y, Zhu Y, Lin J, Zwart A, Wang M, Klimach UM, Wang YJ, Renshaw L, Larionov A, Miller WR, Clarke R (2011). Molecular signaling distinguishes early ER positive breast cancer recurrences despite adjuvant tamoxifen. Cancer Res.

[16] Joensuu KM, Leidenius MH, Andersson LC, Heikkilä PS (2009). High expression of maspin is associated with early tumor relapse in breast cancer. Hum Pathol.

[17] Network CGA (2012). Comprehensive molecular portraits of human breast tumours. Nature.

[18] Yamamoto M, Hosoda M, Nakano K, Jia S, Hatanaka KC, Takakuwa E, Hatanaka Y, Matsuno Y, Yamashita H (2014). p53 accumulation is a strong predictor of recurrence in estrogen receptor-positive breast cancer patients treated with aromatase inhibitors. Cancer Sci.

[19] Bianchini G, Pusztai L, Iwamoto T, Kelly CM, Zambetti M, Fasolo A, Del Conte G, Santarpia L, Symmans WF, Gianni L (2011). Molecular tumor characteristics influence adjuvant endocrine treatment outcome. Cancer Res.

[20] van de Vijver MJ, He YD, van’t Veer LJ, Dai H, Hart AA, Voskuil DW, Schreiber GJ, Peterse JL, Roberts C, Marton MJ, Parrish M, Atsma D, Witteveen A, Glas A, Delahaye L, van der Velde T, Bartelink H, Rodenhuis S, Rutgers ET, Friend SH, Bernards R (2002). A gene-expression signature as a predictor of survival in breast cancer. N Engl J Med.

[21] Buyse M, Loi S, van’t Veer L, Viale G, Delorenzi M, Glas AM, d’Assignies MS, Bergh J, Lidereau R, Ellis P, Harris A, Bogaerts J, Therasse P, Floore A, Amakrane M, Piette F, Rutgers E, Sotiriou C, Cardoso F, Piccart MJ, TRANSBIG Consortium (2006). Validation and clinical utility of a 70-gene prognostic signature for women with node-negative breast cancer. J Natl Cancer Inst.

[22] Rutgers E, Piccart-Gebhart MJ, Bogaerts J, Delaloge S, Veer LV, Rubio IT, Viale G, Thompson AM, Passalacqua R, Nitz U, Vindevoghel A, Pierga JY, Ravdin PM, Werutsky G, Cardoso F (2011). The EORTC 10041/BIG 03-04 MINDACT trial is feasible: results of the pilot phase. Eur J Cancer.

[23] Metzger Filho O, Ignatiadis M, Sotiriou C (2011). Genomic Grade Index: an important tool for assessing breast cancer tumor grade and prognosis. Crit Rev Oncol Hematol.

[24] Metzger-Filho O, Catteau A, Michiels S, Buyse M, Ignatiadis M, Saini KS, de Azambuja E, Fasolo V, Naji S, Canon JL, Delrée P, Coibion M, Cusumano P, Jossa V, Kains JP, Larsimont D, Richard V, Faverly D, Cornez N, Vuylsteke P, Vanderschueren B, Peyro-Saint-Paul H, Piccart M, Sotiriou C (2013). Genomic Grade Index (GGI): feasibility in routine practice and impact on treatment decisions in early breast cancer. PLoS One.

[25] Paik S, Shak S, Tang G, Kim C, Baker J, Cronin M, Baehner FL, Walker MG, Watson D, Park T, Hiller W, Fisher ER, Wickerham DL, Bryant J, Wolmark N (2004). A multigene assay to predict recurrence of tamoxifen-treated, node-negative breast cancer. N Engl J Med.

[26] Paik S, Tang G, Shak S, Kim C, Baker J, Kim W, Cronin M, Baehner FL, Watson D, Bryant J, Costantino JP, Geyer CE, Wickerham DL, Wolmark N (2006). Gene expression and benefit of chemotherapy in women with node-negative, estrogen receptor-positive breast cancer. J Clin Oncol.

[27] Albain KS, Barlow WE, Shak S, Hortobagyi GN, Livingston RB, Yeh IT, Ravdin P, Bugarini R, Baehner FL, Davidson NE, Sledge GW, Winer EP, Hudis C, Ingle JN, Perez EA, Pritchard KI, Shepherd L, Gralow JR, Yoshizawa C, Allred DC, Osborne CK, Hayes DF, Breast Cancer Intergroup of North America (2010). Prognostic and predictive value of the 21-gene recurrence score assay in postmenopausal women with node-positive, oestrogen-receptor-positive breast cancer on chemotherapy: a retrospective analysis of a randomised trial. Lancet Oncol.

[28] Dowsett M, Cuzick J, Wale C, Forbes J, Mallon EA, Salter J, Quinn E, Dunbier A, Baum M, Buzdar A, Howell A, Bugarini R, Baehner FL, Shak S (2010). Prediction of risk of distant recurrence using the 21-gene recurrence score in node-negative and node-positive postmenopausal patients with breast cancer treated with anastrozole or tamoxifen: a TransATAC study. J Clin Oncol.

[29] Gianni L, Zambetti M, Clark K, Baker J, Cronin M, Wu J, Mariani G, Rodriguez J, Carcangiu M, Watson D, Valagussa P, Rouzier R, Symmans WF, Ross JS, Hortobagyi GN, Pusztai L, Shak S (2005). Gene expression profiles in paraffin-embedded core biopsy tissue predict response to chemotherapy in women with locally advanced breast cancer. J Clin Oncol.

[30] Chang JC, Makris A, Gutierrez MC, Hilsenbeck SG, Hackett JR, Jeong J, Liu ML, Baker J, Clark-Langone K, Baehner FL, Sexton K, Mohsin S, Gray T, Alvarez L, Chamness GC, Osborne CK, Shak S (2008). Gene expression patterns in formalin-fixed, paraffin-embedded core biopsies predict docetaxel chemosensitivity in breast cancer patients. Breast Cancer Res Treat.

[31] Cuzick J, Dowsett M, Pineda S, Wale C, Salter J, Quinn E, Zabaglo L, Mallon E, Green AR, Ellis IO, Howell A, Buzdar AU, Forbes JF (2011). Prognostic value of a combined estrogen receptor, progesterone receptor, Ki-67, and human epidermal growth factor receptor 2 immunohistochemical score and comparison with the Genomic Health recurrence score in early breast cancer. J Clin Oncol.

[32] Nielsen TO, Parker JS, Leung S, Voduc D, Ebbert M, Vickery T, Davies SR, Snider J, Stijleman IJ, Reed J, Cheang MC, Mardis ER, Perou CM, Bernard PS, Ellis MJ (2010). A comparison of PAM50 intrinsic subtyping with immunohistochemistry and clinical prognostic factors in tamoxifen-treated estrogen receptor-positive breast cancer. Clin Cancer Res.

[33] Parker JS, Mullins M, Cheang MC, Leung S, Voduc D, Vickery T, Davies S, Fauron C, He X, Hu Z, Quackenbush JF, Stijleman IJ, Palazzo J, Marron JS, Nobel AB, Mardis E, Nielsen TO, Ellis MJ, Perou CM, Bernard PS (2009). Supervised risk predictor of breast cancer based on intrinsic subtypes. J Clin Oncol.

[34] Dowsett M, Sestak I, Lopez-Knowles E, Sidhu K, Dunbier AK, Cowens JW, Ferree S, Storhoff J, Schaper C, Cuzick J (2013). Comparison of PAM50 risk of recurrence score with Oncotype DX and IHC4 for predicting risk of distant recurrence after endocrine therapy. J Clin Oncol.

[35] Jonat W, Gnant M, Boccardo F, Kaufmann M, Rubagotti A, Zuna I, Greenwood M, Jakesz R (2006). Effectiveness of switching from adjuvant tamoxifen to anastrozole in postmenopausal women with hormone-sensitive early-stage breast cancer: a meta-analysis. Lancet Oncol.

[36] Sestak I, Cuzick J, Dowsett M, Lopez-Knowles E, Filipits M, Dubsky P, Cowens JW, Ferree S, Schaper C, Fesl C, Gnant M (2013). Prediction of late distant recurrence after 5 years of endocrine treatment: a combined analysis of patients from the Austrian Breast and Colorectal Cancer Study Group 8 and Arimidex, Tamoxifen Alone or in Combination randomized trials using the PAM50 risk of recurrence score. J Natl Cancer Inst.

[37] Jerevall PL, Ma XJ, Li H, Salunga R, Kesty NC, Erlander MG, Sgroi DC, Holmlund B, Skoog L, Fornander T, Nordenskjöld B, Stål O (2011). Prognostic utility of HOXB13:IL17BR and molecular grade index in early-stage breast cancer patients from the Stockholm trial. Br J Cancer.

[38] Zhang Y, Schnabel CA, Schroeder BE, Jerevall PL, Jankowitz RC, Fornander T, Stål O, Brufsky AM, Sgroi D, Erlander MG (2013). Breast cancer index identifies early-stage estrogen receptor-positive breast cancer patients at risk for early- and late-distant recurrence. Clin Cancer Res.

[39] Sgroi DC, Sestak I, Cuzick J, Zhang Y, Schnabel CA, Schroeder B, Erlander MG, Dunbier A, Sidhu K, Lopez-Knowles E, Goss PE, Dowsett M (2013). Prediction of late distant recurrence in patients with oestrogen-receptor-positive breast cancer: a prospective comparison of the breast-cancer index (BCI) assay, 21-gene recurrence score, and IHC4 in the TransATAC study population. Lancet Oncol.

[40] Filipits M, Rudas M, Jakesz R, Dubsky P, Fitzal F, Singer CF, Dietze O, Greil R, Jelen A, Sevelda P, Freibauer C, Müller V, Jänicke F, Schmidt M, Kölbl H, Rody A, Kaufmann M, Schroth W, Brauch H, Schwab M, Fritz P, Weber KE, Feder IS, Hennig G, Kronenwett R, Gehrmann M, Gnant M, EP Investigators (2011). A new molecular predictor of distant recurrence in ER-positive, HER2-negative breast cancer adds independent information to conventional clinical risk factors. Clin Cancer Res.

[41] Dubsky P, Brase JC, Fisch K, Jakesz R, Singer C, Greil R, Dietze O, Weber KE, Petry C, Kronenwett R, Rudas M, Knauer M, Gnant M, Filipits M (2012). The EndoPredict score identifies late distant metastases in ER+/HER2- breast cancer patients. Cancer Res.

[42] Cheng Q, Chang JT, Gwin WR, Zhu J, Ambs S, Geradts J, Lyerly HK (2014). A signature of epithelial-mesenchymal plasticity and stromal activation in primary tumor modulates late recurrence in breast cancer independent of disease subtype. Breast Cancer Res.

[43] Lianidou ES, Markou A, Strati A (2012). Molecular characterization of circulating tumor cells in breast cancer: challenges and promises for individualized cancer treatment. Cancer Metastasis Rev.

[44] Bidard FC, Mathiot C, Delaloge S, Brain E, Giachetti S, de Cremoux P, Marty M, Pierga JY (2010). Single circulating tumor cell detection and overall survival in nonmetastatic breast cancer. Ann Oncol.

[45] Xenidis N, Markos V, Apostolaki S, Perraki M, Pallis A, Sfakiotaki G, Papadatos-Pastos D, Kalmanti L, Kafousi M, Stathopoulos E, Kakolyris S, Mavroudis D, Georgoulias V (2007). Clinical relevance of circulating CK-19 mRNA-positive cells detected during the adjuvant tamoxifen treatment in patients with early breast cancer. Ann Oncol.

[46] Xenidis N, Perraki M, Kafousi M, Apostolaki S, Bolonaki I, Stathopoulou A, Kalbakis K, Androulakis N, Kouroussis C, Pallis T, Christophylakis C, Argyraki K, Lianidou ES, Stathopoulos S, Georgoulias V, Mavroudis D (2006). Predictive and prognostic value of peripheral blood cytokeratin-19 mRNA-positive cells detected by real-time polymerase chain reaction in node-negative breast cancer patients. J Clin Oncol.

[47] Zhang L, Riethdorf S, Wu G, Wang T, Yang K, Peng G, Liu J, Pantel K (2012). Meta-analysis of the prognostic value of circulating tumor cells in breast cancer. Clin Cancer Res.

[48] Bidard FC, Fehm T, Ignatiadis M, Smerage JB, Alix-Panabières C, Janni W, Messina C, Paoletti C, Müller V, Hayes DF, Piccart M, Pierga JY (2013). Clinical application of circulating tumor cells in breast cancer: overview of the current interventional trials. Cancer Metastasis Rev.

[49] Saloustros E, Perraki M, Apostolaki S, Kallergi G, Xyrafas A, Kalbakis K, Agelaki S, Kalykaki A, Georgoulias V, Mavroudis D (2011). Cytokeratin-19 mRNA-positive circulating tumor cells during follow-up of patients with operable breast cancer: prognostic relevance for late relapse. Breast Cancer Res.

[50] Sestak I, Dowsett M, Ferree S, Baehner F, Cowens JW, Cuzick J (2014). Analysis of molecular score for the prediction of distant recurrence according to body mass index and age at basline. Ann Oncol.

[51] Zhang XH, Giuliano M, Trivedi MV, Schiff R, Osborne CK (2013). Metastasis dormancy in estrogen receptor-positive breast cancer. Clin Cancer Res.

[52] Shah SP, Morin RD, Khattra J, Prentice L, Pugh T, Burleigh A, Delaney A, Gelmon K, Guliany R, Senz J, Steidl C, Holt RA, Jones S, Sun M, Leung G, Moore R, Severson T, Taylor GA, Teschendorff AE, Tse K, Turashvili G, Varhol R, Warren RL, Watson P, Zhao Y, Caldas C, Huntsman D, Hirst M, Marra MA, Aparicio S (2009). Mutational evolution in a lobular breast tumour profiled at single nucleotide resolution. Nature.

[53] Sgroi D, Sestak I, Cuzick J, Zhang Y, Schnabel CA, Erlander MG, Goss PE, Dowsett M (2012). Comparative performance of Breast Cancer Index (BCI) vs. Oncotype Dx and IHC4 in the prediction of late recurrence in hormonal receptor-positive lymph node-negative breast cancer patients: a TransATAC study. Cancer Res.

[54] Bidard FC, Hajage D, Bachelot T, Delaloge S, Brain E, Campone M, Cottu P, Beuzeboc P, Rolland E, Mathiot C, Pierga JY (2012). Assessment of circulating tumor cells and serum markers for progression-free survival prediction in metastatic breast cancer: a prospective observational study. Breast Cancer Res.

[55] Smerage JB, Barlow WE, Hayes D, Winer EP, Leyland-Jones B, Srkalovic G, Tejwani S, Schott AF, O’Rourke MA, Lew DL, Gralow JR, Livingston RB, Hortobagyi GN (2013). SWOG S0500 – a randomized phase III trial to test the strategy of changing therapy versus maintaining therapy for metastatic breast cancer patients who have elevated circulating tumor cell (CTC) levels at first follow-up assessment. Cancer Res.

[56] Bidard FC, Asselain B, Baffert S, Brain E, Campone M, Delaloge S, et al. Use of early CIRculating tumor CElls count changes to guide the use of chemotherapy in advanced metastatic breast cancer patients: the CirCe01 randomized trial. Cancer Res 2013;72 Suppl 3.

